# Kernohan's Notch: A Forgotten Cause of Hemiplegia—CT Scans Are Useful in This Diagnosis

**DOI:** 10.1155/2013/296874

**Published:** 2013-11-20

**Authors:** Ragesh Panikkath, Deepa Panikkath, Sian Yik Lim, Kenneth Nugent

**Affiliations:** Department of Internal Medicine, Texas Tech University Health Sciences Center, Lubbock, TX 79430, USA

## Abstract

Hemiparesis ipsilateral to a cerebral lesion can be a false localizing sign. This is due to midline shift of the midbrain resulting in compression of the contralateral pyramidal fibers on the tough dural reflection tentorium cerebelli. This may result in partial or complete damage to these fibers. Since these fibers are destined to cross in the medulla and innervate the opposite side of the body, this causes hemiparesis ipsilateral to the site of cerebral lesion. Computed tomography (CT) scans have not been used to support the diagnosis of this entity until now. We report a 68-year-old woman with a subdural hematoma who developed ipsilateral hemiparesis without any other explanation (Kernohan's notch). The CT of the head showed evidence of compression of the midbrain contralateral to the hematoma and was useful in the diagnosis. The purpose of this report is to increase the awareness of this presentation and to emphasize the utility of CT scans to support the diagnosis.

## 1. Introduction

Hemiparesis ipsilateral to the site of a cerebral lesion has been called Kernohan's notch. This sign (originally described at autopsy) has been reported with cerebral tumors, subdural hematomas (SDH), and extradural hematomas with midline shift. Recently magnetic resonance imaging (MRI) has been used for this diagnosis in appropriate clinical situations; indirect supportive evidence for this sign has not been described utilizing the more commonly available computed tomography (CT) imaging.

## 2. A Case Report

**Figure 1:**
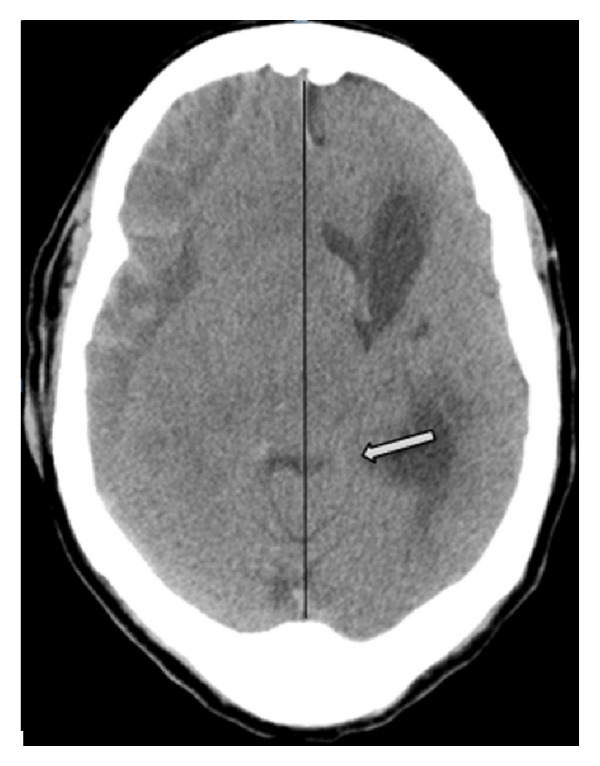
CT of the head showing gross posterior and lateral displacement of midbrain with deformity of the crus cerebri (pointed by the arrow) to the left side. The midline is marked with a black line. The subdural hematoma is visible on the right-side.

**Figure 2:**
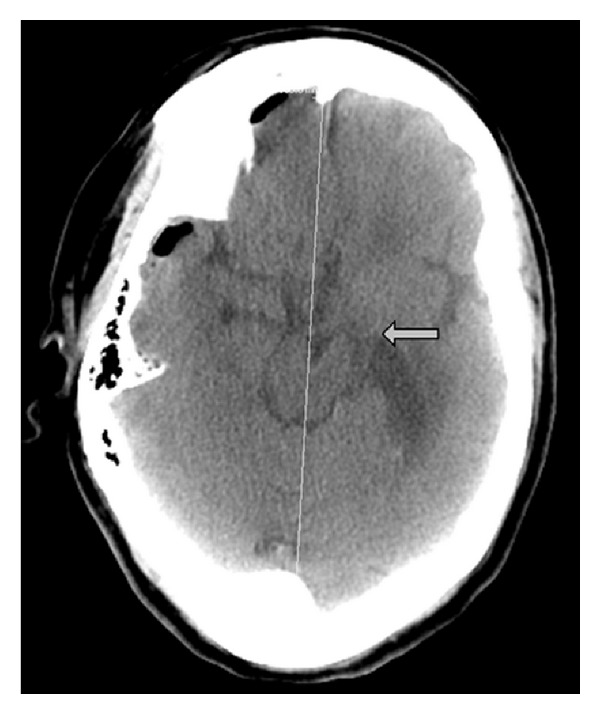
CT of the head after craniotomy and evacuation of subdural hematoma showing persistent midline shift of the midbrain with deformity of crus cerebri (marked by arrow).

We report a 69-year-old woman with Alzheimer's disease and recurrent falls who presented in a comatose state. A CT scan of the head showed a large right subdural hematoma with a midline shift of 18 mm. She underwent urgent neurosurgical evacuation of the subdural hematoma. Her consciousness gradually recovered, but she had right hemiplegia. An MRI did not show any left-sided infarcts which would explain this. Since her hemiplegia was ipsilateral to the side of the subdural hematoma, the possibility of Kernohan's notch was considered. Gross deviation of the crura of the midbrain to the left side was noted on detailed review of the CT scan of the head done prior to the evacuation of the hematoma (Figure 1). A CT of head after craniotomy and evacuation of subdural hematoma showed persistent deformity of the midbrain (Figure 2). The MRI images also showed evidence of a shift of the midbrain to the left with hyperintensity in the midbrain in the region of compression. The clinical picture of ipsilateral weakness in a patient with a subdural hematoma, evidence of compression of the midbrain in the CT and MRI of the brain, and the absence of any infarcts on the contralateral side in diffusion weighted T1 and T2 sequences of MRI confirmed our suspicion of Kernohan's notch.

## 3. Discussion

The corticospinal tracts originate in the motor cortex in the frontal lobes and descend through the internal capsules and subsequently through the midbrain and pons, before the majority of the fibers (80%) decussate in the medulla. Due to this decussation, the left cerebral motor cortex controls the movements of the right-side of the body and vice versa. In the midbrain, the pyramidal tract courses anteriorly in the crus cerebri. Due to the crossing of pyramidal tract fibers downstream in the medulla, damage of the fibers in the midbrain results in paralysis of the opposite side of the body. The pyramidal tract controls discrete voluntary movements, like skilled and precise movements.

**Figure 3:**
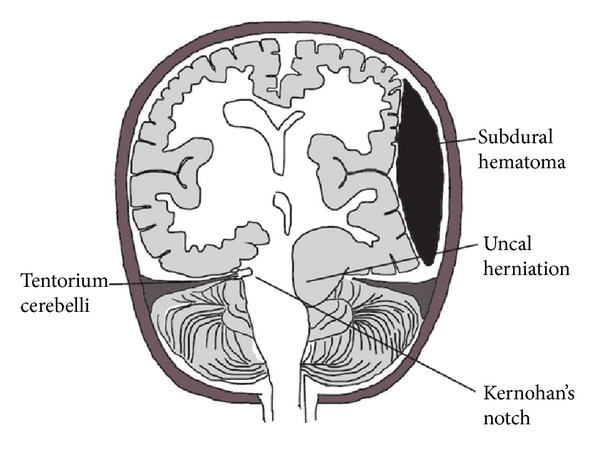
Schematic representation of Kernohan's notch. Demonstrated here are a subdural hematoma and uncal herniation on the same side. Notching of the midbrain is seen on the opposite side (Kernohan's notch). This damages the contralateral pyramidal tract fibers in the midbrain and causes hemiparesis on the side of subdural hematoma.

The explanation for ipsilateral hemiparesis to the side of a space occupying lesion predates the invention of MRI and CT imaging techniques. Kernohan and Woltman in an autopsy study reported the presence of a notch in the midbrain contralateral to the side of the space occupying lesion due to compression of the midbrain against tough dural reflection tentorium cerebelli [1, 2] as shown in the schematic representation shown in Figure 3. This can damage the crus cerebri of the midbrain (carrying pyramidal fibers) resulting in complete, partial, or no disruption of the pyramidal fibers located in it. Since the pyramidal fibers in the midbrain innervate the opposite side of the body, it results in paralysis ipsilateral to the site of the lesion. The recovery after hemiplegia may be complete, partial, or none depending on the extent of damage to pyramidal fibers. This phenomenon has been described with cerebral tumors, extradural hematoma, and subdural hematomas.

The recognition of this syndrome is critically important; if unrecognized, it may lead to surgery on the wrong side of the brain. For example, 80 years after the description of this phenomenon by Kernohan, Wolf reported an unfortunate incident in which a patient presented with a subdural hematoma after getting hit by a golf stick [3]. The patient had left-sided weakness, and the CT of the head demonstrated a left-sided SDH. The surgeons thought that the left-right markers of the CT scan image were misplaced. Two burr holes followed by a craniotomy on the right-side did not reveal any SDH. A CT of the head performed the next day showed the presence of a right-sided craniotomy and a left-sided SDH.

 Kernohan's notch phenomenon has been previously demonstrated by MRI. An abnormal signal in T2-weighted images on MRI in the contralateral midbrain may be a marker for poor neurological recovery [4]. CT scans have not been thought to be helpful in diagnosing this condition. This is the first report of a CT scan being used to support the diagnosis of this syndrome. The CT scan obtained for the diagnosis of an intracranial space occupying lesion can identify contralateral midbrain compression as well. This along with clinical features suggestive of ipsilateral pyramidal tract involvement can support a diagnosis of Kernohan's notch. Use of a CT of the head for this purpose does not require additional imaging and is helpful in centers which do not have access to MRI. Above all, awareness of this syndrome and a high index of clinical suspicion are crucial for the diagnosis of Kernohan's notch. 

 The concept of functional deterioration of a focus distant to the site of injury and its role in functional recovery after a stroke was proposed more than a decade before Kernohan published his series of patients. Constantin von Monakov coined the term diaschisis (Greek for shocked throughout) in 1914; this term is used for sudden loss of function of a portion of the brain connected to a distant damaged area. The site of diaschisis and the originally damaged area are connected to each other by neurons, or the damage could arise because of disturbances in regional blood flow [5]. Therefore, damage to a structure can affect the function of remaining intact systems. Monakov proposed that there is a delicate balance between different components of the brain and an injury to a component could affect other parts of the brain even if it is not anatomically damaged or close to the site of injury. 

## 4. Conclusion

In summary, paralysis ipsilateral to the site of a lesion in the brain could be due to compression of the contralateral cerebral peduncles against the tough dural reflections causing damage to the pyramidal tract fibers in the brain stem. This false localizing sign needs to be recognized to prevent inappropriate surgery and medical management. The purpose of this case report is to increase the awareness of this sign and to promote the usefulness of CT scans to support this diagnosis. If this phenomenon is suspected, additional cuts obtained at the midbrain level with 3D reconstruction might be helpful for the diagnosis. More studies are needed to determine if procedures like early surgery (evacuation of hematoma/surgeries aimed at intracranial pressure reduction) in patients with clinical findings consistent with Kernohan's notch will help reduce the compression of the midbrain and its consequences.
